# A Case of Esophago-Respiratory Fistula due to Inhalation Smoke Injury Diagnosed by Upper Endoscopy

**DOI:** 10.1155/2023/4231287

**Published:** 2023-01-09

**Authors:** Aida Nasirishargh, Bao Sean Huy Nguyen, Michael J. Lawson, Eric J. Mao

**Affiliations:** ^1^University of California Davis, School of Medicine, Sacramento 95817, CA, USA; ^2^Division of Gastroenterology and Hepatology, Department of Internal Medicine, University of California Davis Medical Center, Sacramento 95817, CA, USA

## Abstract

Esophago-respiratory fistula (ERF) refers to the formation of a pathological connection between the esophagus and respiratory tract. Acquired ERF is a rare but life-threatening diagnosis in adults. We describe a 79-year-old male who was admitted with an inhalation smoke injury. He was diagnosed with ERF by endoscopic visualization and sampling of the hyaline cartilage within the wall of the esophagus. Percutaneous endoscopic gastrostomy placement and conservative measures were effective in the management of ERF.

## 1. Introduction

Esophago-respiratory fistula (ERF) refers to the formation of a pathological connection between the esophagus and respiratory tract. Although rare in adults, it can cause significant morbidity and mortality in patients [1, 2]. Malignancy, trauma, and infections are the most common causes of the formation of these fistulas [1–5]. Tissue damage from intubation and endoscopic interventions, foreign body ingestions such as taco shells, and blunt chest injuries have been reported in the literature as traumatic events that cause ERF [4, 6, 7]. However, inhalation injury in burn patients is rarely reported as the underlying cause of ERF [8, 9]. Here, we are describing a patient who was admitted with an inhalation smoke injury and was found to have an ERF.

## 2. Case Report

**Figure 1:**
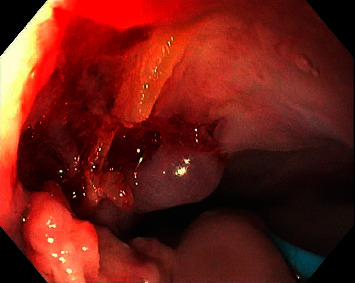
Yellow-tan foreign body in the anterior aspect of the esophagus with surrounding granulation tissue.

A 79-year-old male was initially admitted for cardiac arrest after being rescued from a house fire. The hospital course was notable for intubation for smoke inhalation injury, confirmed by bronchoscopic findings of inflammation including moderate erythema, carbonaceous deposits, and bronchorrhea [10]. On the 30^th^ day of admission, the patient developed acute melena and anemia while on a heparin drip for atrial fibrillation. Vitals were remarkable for hypotension and tachycardia, and his hemoglobin level was 6.5 g/dL. Esophagogastroduodenoscopy (EGD) was remarkable for a midesophageal ulcer and associated mucosal tear surrounded by granulation tissue. On the anterior wall of the esophagus, there was a yellow-tan foreign body that appeared to be embedded onto the esophageal wall (Figure 1). The initial impression of the foreign body was a hard food material such as a taco shell that had been ingested before the house fire and had become firmly embedded within the esophageal wall. The midesophageal ulcer and associated mucosal tear due to the embedded foreign body were likely the sources of the patient's anemia and melena. Two small biopsy samples of the foreign body were sent for pathological analysis.

**Figure 2:**
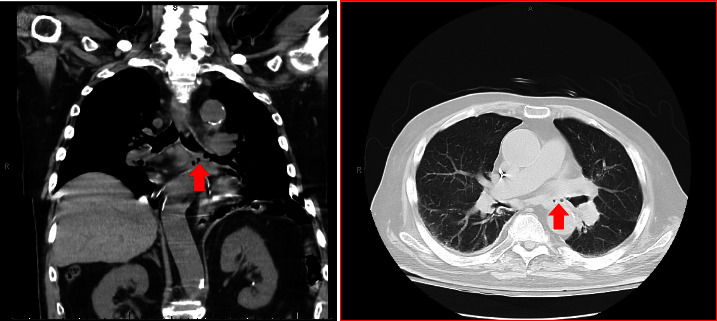
CT image without contrast of the patient's chest. Arrows indicating extraluminal air tracking from the left mainstem bronchus towards the esophagus.

Given the midesophageal ulcer and projected need for prolonged nasogastric tube feeding due to dysphagia, a percutaneous endoscopic gastrostomy (PEG) tube was placed the following day. Eventually, the pathology of the foreign body samples revealed fragments of hyaline cartilage, similar to that found in the tracheobronchial tree, which was concerning for ERF. Chest CT scan without contrast showed a narrowed left mainstem bronchus abutting the esophagus with foci of suspected extraluminal air along the left lateral margin of the esophagus (Figure 2). This was concerning for an esophageal injury or tear with localized perforation and possible bronchial injury. Therefore, the suspicion was high that the patient had an ERF, likely from the initial inhalation smoke injury. A confirmatory esophagogram was not performed due to the patient's dysphagia and given the high likelihood of an ERF with the EGD and imaging findings. Bronchoscopy was performed 2 weeks after the initial EGD to assess for a true fistulous connection between the left bronchus and esophagus. Bronchoscopy revealed extrinsic compression of the left mainstem bronchus without mucosal defects or obvious fistulous connection to the esophagus. However, there was diffusely inflamed left-sided mucosa and extensive purulent distal mucous plugging, suggestive of prior fistulous connection. This was followed by the placement of a stent in the left mainstem bronchus.

The fistula had either resolved by the time of bronchoscopy or was too small to be visible during the procedure. The follow-up chest CT scan 11 days after stent placement no longer visualized the previously seen foci of air between the esophagus and left mainstem bronchi.

## 3. Discussion

Acquired ERF is a rare but life-threatening diagnosis in adults [4]. A study of patients with ERF who were diagnosed between 2001 and 2011 showed those with benign ERF had a median survival of 74 months [11]. ERF can present as a late complication of thermal inhalation injury. The incidence of inhalational injury in burn patients who required hospitalization ranged from 20% to 30%, and the risk of mortality was increased by 24 times in this population [12, 13]. Mucosal edema along with hypotension and shock in burn patients may compromise the perfusion of the upper airway mucosa, leading to ERF formation. Prolonged intubation and tracheostomy can also be contributing factors for the development of ERF in patients with inhalation injury, especially in those with high cuff pressure, infection, hypotension, steroid use, diabetes, and the use of a nasogastric tube [14].

Although the initial symptoms of ERF can be insignificant, a delayed diagnosis can lead to severe complications such as pneumonia, life-threatening hemoptysis, and respiratory failure [4, 6, 15]. In intubated patients, increased secretions, pneumonia, and evidence of aspiration of gastric contents are concerning for ERF formation [15]. Bronchoscopy and esophageal endoscopy are first-line diagnostic and therapeutic modalities [16]. Although EGD or bronchoscopy may not identify the fistula orifice, like in our case, they may reveal inflammatory changes in the luminal mucosa suggestive of a prior or current fistula [17]. Plain radiography and CT scan of the chest can be helpful with diagnosis if there is evidence of pneumomediastinum or an obvious mucosal tear [17]. An esophagogram with ingestion of barium contrast can be used to confirm the diagnosis [4, 6, 16, 17]. Gastrografin is not recommended due to the risk of acute pulmonary edema and respiratory failure associated with aspiration of it.

The goal of treatment of ERF is to prevent severe complications and optimize nutritional status [16]. Surgical interventions for the closure of fistulas have been recommended for certain cases, especially in large and traumatic ERF [14, 16]. There was no significant difference in survival between surgical and nonsurgical treatment of ERF such as airway or esophageal stent placement in those with nonmalignant ERF [11]. Conservative treatment options such as removing nasogastric tubes and placing gastrostomy or jejunostomy feeding tubes have also been shown to aid the healing of ERF [14].

In summary, we present a case of ERF due to inhalation burn injury, which was diagnosed by endoscopic visualization and sampling of hyaline cartilage within the wall of the esophagus. The most likely cause of ERF was inflammation and irritation of the bronchial mucosa secondary to smoke inhalation. PEG placement and conservative measures were effective in the management and healing of ERF. As in our case, the collaboration between pulmonology and gastroenterology services is essential for the diagnosis and management of this condition.

## Data Availability

The data used to support the findings of this study are available from the corresponding author upon reasonable request, for protecting the patient's identity.
